# Supplementary material for: Multidimensional encoding of restricted and anisotropic diffusion by double rotation of the q vector
Source: Magn Reson (Gott). 2023 Mar 15;4(1):73–85. doi: 10.5194/mr-4-73-2023 (PMC10583292; doi:10.5194/mr-4-73-2023)
Supplement: The supplement related to this article is available online at: https://doi.org/10.5194/mr-4-73-2023-supplement. [file mr-4-73-supplement.zip › mr-4-73-2023-supplement-title-page.pdf]

Supplement of Magn. Reson., 4, 73–85, 2023
https://doi.org/10.5194/mr-4-73-2023-supplement
© Author(s) 2023. CC BY 4.0 License.

Open Access
MAGNETIC RESONANCE

Supplement of

Multidimensional encoding of restricted and anisotropic diffusion by double rotation of the q vector

Hong Jiang et al.

Correspondence to: Daniel Topgaard (daniel.topgaard@fkem1.lu.se)

- mr-4-73-2023-supplement-title-page.pdf
- supplement_fig2code.m

The copyright of individual parts of the supplement might differ from the article licence.
